# Clinical Effect of Electroacupuncture on Acute Sleep Deprivation and Event-Related Potential Affecting the Inhibition Control of the Brain: Study Protocol for a Randomized Controlled Trial

**DOI:** 10.3389/fneur.2022.911668

**Published:** 2022-07-08

**Authors:** Haiping Li, Mengyu Wang, Yiming Wu, Xinwang Chen, Cong Xue, Peidong Liu, Run Zhang, Ziyun Liao

**Affiliations:** ^1^College of Acupuncture, Moxibustion and Tuina, Henan University of Traditional Chinese Medicine, Zhengzhou, China; ^2^School of Rehabilitation Medicine, Henan University of Chinese Medicine, Zhengzhou, China; ^3^The Third Affiliated Hospital of Henan University of Chinese Medicine, Zhengzhou, China

**Keywords:** electroacupuncture, cognition, acute sleep deprived, Go/No-Go, ERP

## Abstract

**Background:**

Acute sleep deprivation (ASD) can effect mood, attention, memory, alertness and metabolism. Especially, it is often accompanied by cognitive impairment of the brain. Acupuncture is safe and effective for improving cognitive function, but its underlying mechanism is not fully understood. In this study, an event-related potential (ERP) technique will be employed to measure the behavioral, cognitive, and physiological changes produced by electroacupuncture intervention after ASD.

**Methods:**

We will recruit 60 healthy subjects. The participants will be randomly divided into a treatment group, a control group, a sham electroacupuncture group and a blank group, at a 1:1:1:1 ratio. The primary outcome will be determined by the change from baseline to 36 h in the MoCA score. The secondary results include the amplitude and latency of ERP N2 and P3, Go-hit rates, Go-RTs, No-Go-FA rates, the WCST, the Digit Span Subtest of the WAIS, the ESS score and FS-14. The 15 healthy subjects will not receive acupuncture treatment and ASD, but will receive EEG records and cognition functions test at the beginning and end of the experiment. Electroacupuncture intervention will be performed for 30 min once every 12 h, a total of three times. ERP measurements and other tests will be performed after baseline and ASD, and the statistician and outcome evaluator will be blinded to treatment allocation.

**Discussion:**

This study is expected to investigate the effectiveness of electroacupuncture in improving cognition for ASD.

**Trial Registration:**

ChiCTR2200055999.

## Background

Acute sleep deprivation (ASD) is the elimination of sleep for a period of time (at least 24 h) to significantly prolong wakefulness (1). Inadequate sleep and sleep disorders have become an important public health problem. ASD can effect mood, attention, memory, alertness, cognitive performance and metabolism (2, 3). With the increasing pace of life, the incidence of sleep deprivation has increased significantly, which has attracted attention in the military field and many social sectors (such as aviation, navigation, medical treatment, and transportation). The common negative effects of sleep deprivation include feeling too sleepy during the day, accidents from lack of attention, mood changes, and changes in appetite (4). Insufficient sleep increases the risk of human error-related accidents (5). ASD affects cognition in many ways and can negatively impact alertness, learning, memory, and executive function (6–12). Since ASD has serious effects on human cognitive brain function, safety intervention studies on the effects of ASD, such as acupuncture, are increasingly popular in this field (13, 14).

Studies show that administration of caffeine may improve vigilance, alertness, mood and cognitive processes and enhance cognitive processing related to response selection and inhibition (15, 16). Compared with the central nervous system stimulant caffeine, acupuncture is a safe alternative therapy with minimal side effects (17). A Delphi expert consensus survey (18) shows that more than 80% of experts agree that acupuncture can be used to improve cognitive function, which has been underpinned by the results of previous studies (19, 20). Although acupuncture has been studied for a long time, there is still a lack of knowledge of the effects of acupuncture on the human brain. More research is necessary to better understand how brain activity is affected by acupuncture.

Executive function (also called executive control or cognitive control) is fundamental to human cognition (21). It is a top-down mental process needed when one has to concentrate and pay attention, when going on automatic or relying on instinct would be insufficient (22–24). There are three core executive functions: inhibition, working memory (WM), and cognitive flexibility (25, 26). The Go/No-go paradigm has been used to study the executive function (27). Event-related potential (ERP) has often been used to investigate the effects of sleep deprivation on neurocognitive functioning. It is non-invasive and simple to perform, and it does not entail discomfort for participants. Certain components of ERP have been found to reflect specific forms of information processing related to sensory, motor and/or cognitive functions (28). The visual Go/No-go task is often used to study response inhibition (29). There have been several reports supporting the hypothesis that the visual N2 reflects a frontal inhibition mechanism (30, 31), including cognitive control and response inhibition (32, 33). Furthermore, recent studies suggest that P3 may play an important role in the post-response stage, reflecting processes of cognitive processing, such as stimulus identification and evaluation (34, 35) or monitoring of inhibition (36, 37).

This high-quality randomized controlled trial (RCT) was designed *via* a pragmatic trial approach to objectively assess the efficacy of electroacupuncture for brain cognition using ERP. By comparing ERP related to response inhibition tasks before and after ASD, we can understand the effect of ASD on the brain's inhibition control and the effect of electroacupuncture on brain cognition function after ASD.

### Objectives

The aim of this study is to assess the effect of electroacupuncture on brain inhibition control function.

## Methods/Design

### Study Setting

This study is a participant-, statistician-, and assessor-blinded parallel randomized control clinical trial. The study will follow the principles of the Consolidated Standards of Reporting Trials (CONSORT) as well as the Standards for Reporting Interventions in Clinical Trials of Acupuncture (STRICTA) statement for acupuncture. Sixty healthy college students will be randomly assigned to a treatment group, a sham electroacupuncture group and a blank group, at a 1:1:1:1 ratio. The control group will be set to evaluate the efficacy of electroacupuncture. The sham electroacupuncture group be set to rule out placebo effects. The study will be conducted at the Third Affiliated Hospital of Henan University of Traditional Chinese Medicine, Department of Acupuncture, Henan, China, following the Standard Protocol Items: Recommendations for Interventional Trials (SPIRIT). For the participant timeline, see Table 1.

**Table 1:** SPIRIT figure for schedule of enrollment, interventions and assessments.

	**Study Period**						
	**Enrollment**	**Allocation**	**Post-allocation**				**Close-out**
**Timepoint**	***−1 w***	**0**	***Baseline***	***12 h***	***24 h***	***36 h***	***48 h***
**Enrollment**							
Eligibility screen	X						
Informed consent	X						
Baseline assessment	X						
Allocation		X					
**Interventions**							
Treatment group			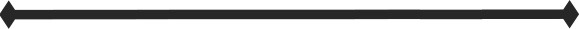				
Placebo group			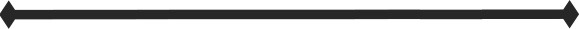				
Control group			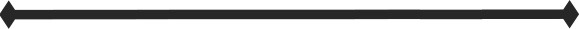				
Blank group							
**Assessments**							
EHQ	X						
Sleep log	X						
SCL-90	X						
PSQI	X						
MoCA			X			X	
WCST			X			X	
DS			X			X	
ESS			X			X	
FS-14			X			X	
EEG-record			X			X	
Adverse events			X	X	X	X	X

*EHQ, Edinburgh Handedness Questionnaire; SCL-90, Symptom Checklist-90; PSQI, Pittsburgh Sleep Quality Index; MoCA, Montreal Cognitive Assessment; WCST, Wisconsin Card Sorting Test; DS, Digit Span of the Wechsler Adult Intelligence Scale; ESS, Epworth Sleepiness Scale; FS-14, Fatigue Scale-14; EEG, Electroencephalogram*.

### Sample Size

According to the pre-test study, the average MoCA scores in the treatment group, control group, and blank group are 26.70, 25.72, and 27.32, respectively, and the standard deviations are 1.03, 1.59, and 1.37, respectively. Using the sample size calculation method of multi-group mean, the sample size estimation [one-way analysis of variance (ANOVA)] *F*-test] method will use the test level α = 0.05 (bilateral) and test efficiency 1 – β = 0.90. The bilateral test will be used. The total sample size of 45 cases was estimated using PASS 15.0 software.

## Recruitment

All participants will be recruited through advertisements on websites and posters at colleges and universities near Henan University of Traditional Chinese Medicine. Treatment and measurements will be performed in quiet rooms. Participants will contact the recruitment staff by telephone or WeChat. Recruitment staff will be responsible for the enrollment of participants. If they meet the study criteria, they will be invited to the study. Eligible candidates will be asked to sign an informed consent form before the experiment begins. We will provide accommodation and meals during the experiment, and provide the participants a certain reward at the end of the experiment.

## Inclusion Criteria

Recruitment conditions are as follows: (1) male college student in good health after physical examination; (2) 18–24 years of age and right-hand according to the modified Edinburgh Handedness Questionnaire; (3) normal or corrected-to-normal visual acuity; (4) normal state of sleep awakening, with no unusual sleep schedule–that is, waking up unusually early or late–and no history of shift; the self-made “sleep log” table and interview show that the subjects have good sleep habits; (5) on Symptom Checklist 90 (Symptom List-90 SCL Mel 90), the total average score is <1, and the score of each factor is <1; (6) the score of the Pittsburgh Sleep Quality Index (PSQI) is <5; (7) no habit of drinking coffee, tea, or alcohol or smoking; (8) voluntarily signing an informed consent form. The participants will be contacted a few days later to determine whether they are interested in participating, and, if so, an appointment will be made for them to physical examination package at the Third Affiliated Hospital of Henan University of Traditional Chinese Medicine.

## Exclusion Criteria

Participants with any of the following conditions will be excluded: (1) having taken sedative or sleep-aiding drugs, such as estazolam or alprazolam, in the past month; (2) having received acupuncture or moxibustion treatment in the past month; (3) severe heart, liver, or kidney disease, mental illness, or coagulation dysfunction; (4) family history of mental illness or history of infectious diseases; (5) allergies, especially to needles; (6) implantation of a cardiac pacemaker or implantable electronic equipment.

## Dropout Criteria

(1) Those who do not fill in the cases in a timely and accurate manner or those who do not fill in the cases properly; (2) those who fail to follow the plan for treatment in the course of the trial; (3) subjects who automatically asked to withdraw.

## Randomization

Unrestricted (simple) randomization will be used to allocate participants to either the treatment group, control group, blank group. Participants will be randomized using sequentially numbered, opaque sealed envelopes (SNOSE) to maintain allocation concealment (38). Different personnel will carry out tasks such as assigning a sequence, recruiting subjects, or intervention.

## Blinding

In this trial, outcome assessors, and data analysts will be blinded to the treatment allocation to minimize potential sources of bias and the clinical researcher, assessor, and statistician will not share study information with each other. After the end of the experiment, we will use a questionnaire to ask the participants if they know whether they received real acupuncture or sham acupuncture.

## Intervention

All practitioners in this trial are licensed TCM acupuncture therapists with at least 5 years of clinical experience, and they will be trained to master the study protocol. The acupuncturist will be asked to administer the standard manipulation.

## Treatment Group

The treatment group will receive electroacupuncture and 36-h ASD. The choice of acupuncture points is based on a previous study (18). The acupoints will be Baihui (GV20), Sishencong (EX-HN1), and Shenting (GV24). Acupuncture at these acupoints mentioned above can regulate Qi of Governor Vessel, clear the mind, lift the spirits, nourish Yang based on Chinese acupuncture theory (39). Modern research shows that Baihui, Shenting and sishencong acupuncture can alleviates cognitive impairment (40–42).We will use sterile, disposable stainless-steel needles of the Hwato brand measuring 0.30 × 25 mm, with horizontal needling of 12–20 mm following an angle of 0–15°. After eliciting the Deqi response, the researcher will apply electroacupuncture by connecting an acupoint nerve stimulator (G6805-2A) to Baihui (GV20) and Shenting (GV24). The stimulation parameters are dense wave, frequency of 5 Hz, current of 1–5 mA, and needle retention for 30 min, with intervention performed every 12 h.

## Placebo Group

Placebo group is intervened with sham electroacupuncture. The sham GV20 point is 0.5 cun (≈12.5 mm) lateral to GV20, the sham EX-HN1 point is 0.5 cun (≈12.5 mm) horizontal to EX-HN1 and the sham GV24 point is 0.5 cun (≈12.5 mm) horizontal to GV24, pierce of 3–5 mm following an angle of 0–15°. Procedures, electrode placements, and other treatment settings are the same as in the treatment group but with no electricity output and needle manipulation for de qi.

## Control Group

Participants in the control group will receive only 36-h ASD but will not receive electroacupuncture treatment.

## Blank Group

The healthy control group will not receive any intervention. They will keep normal work and rest time.

## Visual Go/No-Go Task Design

At the beginning of the visual Go/No-Go task, a small white cross (+) on a black background appears at the center of the screen for 100 ms before each trial, followed by the stimulus. Each stimulus is presented for a duration of 100 ms, with an inter-stimulus interval of 800 ms. The task has two blocks with 150 trials in each block. In one block, the subjects are asked to respond to the left arrow [target stimulus (Go)] and withhold responding to the right arrow [non-target stimulus (No-Go)], while in the other block, the response pattern is reversed. The Go stimuli occur with a one-third probability, and the sequence of Go/No-Go stimuli is pseudorandom to ensure that the No-Go stimuli do not appear in a continuous sequence. The participants will undergo a training session to ensure that they understand the Go/No-Go task and that their performance is above 90%.

**Figure 1:**
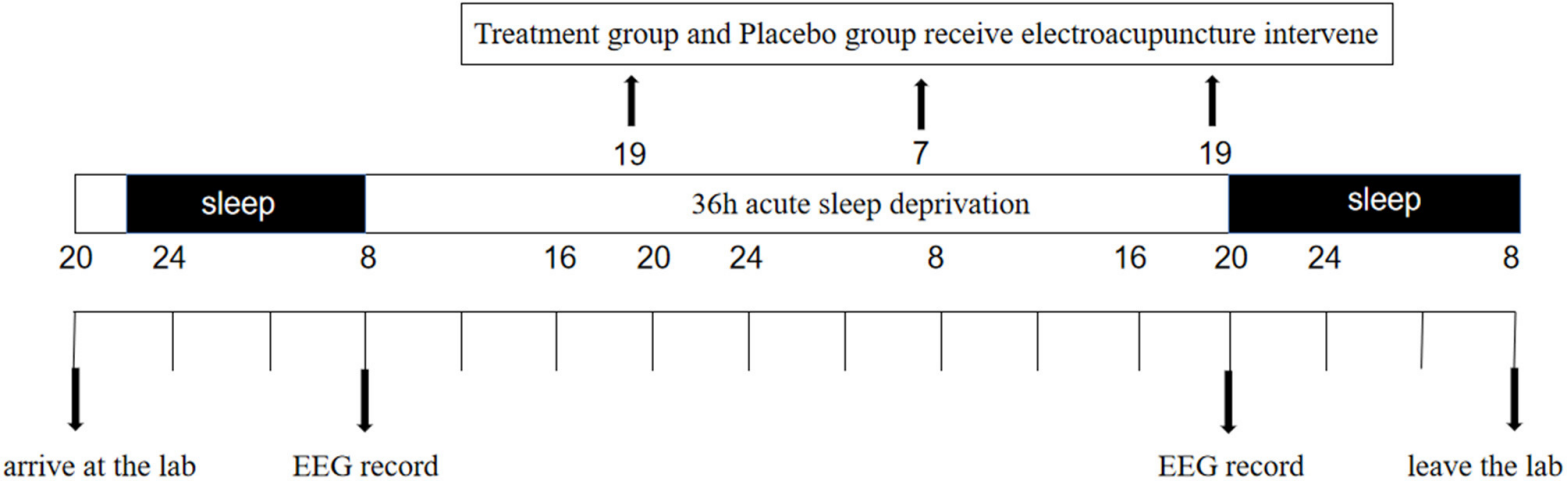
Study protocol. In the study protocol, subjects will undergo 36 h periods of acute sleep deprivation (ASD). The black arrows indicate the time points of Electro- encephalogram (EEG) recordings and treatment group receive electroacupuncture treatment.

The participants will arrive at the lab at 20:00 p.m. and perform a Go/No-Go task as a baseline. They will be asked to ensure that they get a full night's sleep (by wearing a wrist-operated sleep monitor and “sleep log” to ensure that they get at least 8 h of sleep), and the experiment will begin at 8:00 a.m. the next morning, taking the Go/No-Go task as 0 h. The participants will not be allowed to sleep for the next 36 h, during which time they will receive acupuncture every 11 h. After 36 h, the participants will be asked to complete the Go/No-Go task again. They will be accompanied and supervised by 10 staff members, and there will be two emergency doctors and nurses in the laboratory at all times. The participants will be asked to stay in the laboratory and will be allowed to talk, read, play computer games, and engage in other non-strenuous activities. They will not be allowed to smoke or drink coffee, tea, hot chocolate, wine, or other bladder-irritating drinks (Figure 1).

## EEG Recording and ERP Analysis

The subjects will sit comfortably in a quiet room. Continuous EEG recordings will be obtained using Brain Vision recorder software. The sampling frequency will be 1,000 Hz, and the electrode impedances will be maintained below 5 kΩ. The electrodes will be placed according to the international 10–20 system; a total of 32 electrodes will be recorded in this study. ERP component analysis will be performed using Matlab 2018b and will include amplitudes and latencies. EEG data will be analyzed and collected by people who have received professional training.

## Outcomes

### Primary Outcome

The primary outcome is the MoCA score. The cognitive areas assessed include attention and concentration, executive function, memory, language, visual structure skills, abstract thinking, calculation and orientation, with a total score of 30. The lower the score, the more severe the cognitive impairment.

### Secondary Outcomes

The behavioral data and latency and amplitude of N2 and P3 amplitude will be used to evaluate the effect of sleep deprivation and the intervention effect of acupuncture. ERP component detection, such as N2 and P3, has been used for the measurement of brain activity (13) and as a reflection of brain executive function (30–37). The behavioral outcome variables include the mean RT for correct hits, hit rates (correct button presses for Go stimuli), and the percentage of false alarms (FAs, incorrect button presses in response to No-Go stimuli), which are used as indices of individual behavior performance. Executive function and working memory will be assessed with the Wisconsin Card Sorting Test (WCST). Working memory and the Digit Span (DS) of the Wechsler Adult Intelligence Scale (43). The Epworth Sleepiness Scale (ESS), also known as the Epworth Daytime Sleepiness Scale, developed by Johns (44), will be used to evaluate excessive drowsiness during the day. The maximum score on the scale is 24 points, with >6 points indicating drowsiness, >11 points indicating excessive drowsiness, and >16 points indicating dangerous drowsiness. The FS-14 was jointly compiled by a number of experts, including Chalder and Berelowitz, in the United Kingdom (45). The physical fatigue score is obtained by adding the scores of eight items (1–8), the mental fatigue score is obtained by adding the scores of six items (9–14), and the total fatigue score is the sum of physical and mental fatigue scores. The highest score of physical fatigue is 8, the highest score of mental fatigue is 6, and the highest total score is 14. The higher the score, the more serious the fatigue.

### Other Outcomes

Participant characteristics such as age, weight, body mass index, blood pressure, heart rate, pulse, liver function, renal function, electrocardiogram, and back pain history will be collected using electronic case report forms.

### Adverse Events

Any adverse event resulting from electroacupuncture in participants, such as headache, nausea, dizziness, localized infections, bleeding, or local subcutaneous hematoma, will be recorded, assessed, and reported. The occurrence time, cessation time duration, correlation with acupuncture, measures taken, and outcomes of adverse events will also be recorded. If serious adverse events occur, the Institutional Ethics Committee of the Third Affiliated Hospital of Henan University of Traditional Chinese Medicine will be informed, and the committee will decide whether to unblind and withdraw from the study.

### Follow-Up

**Figure 2:**
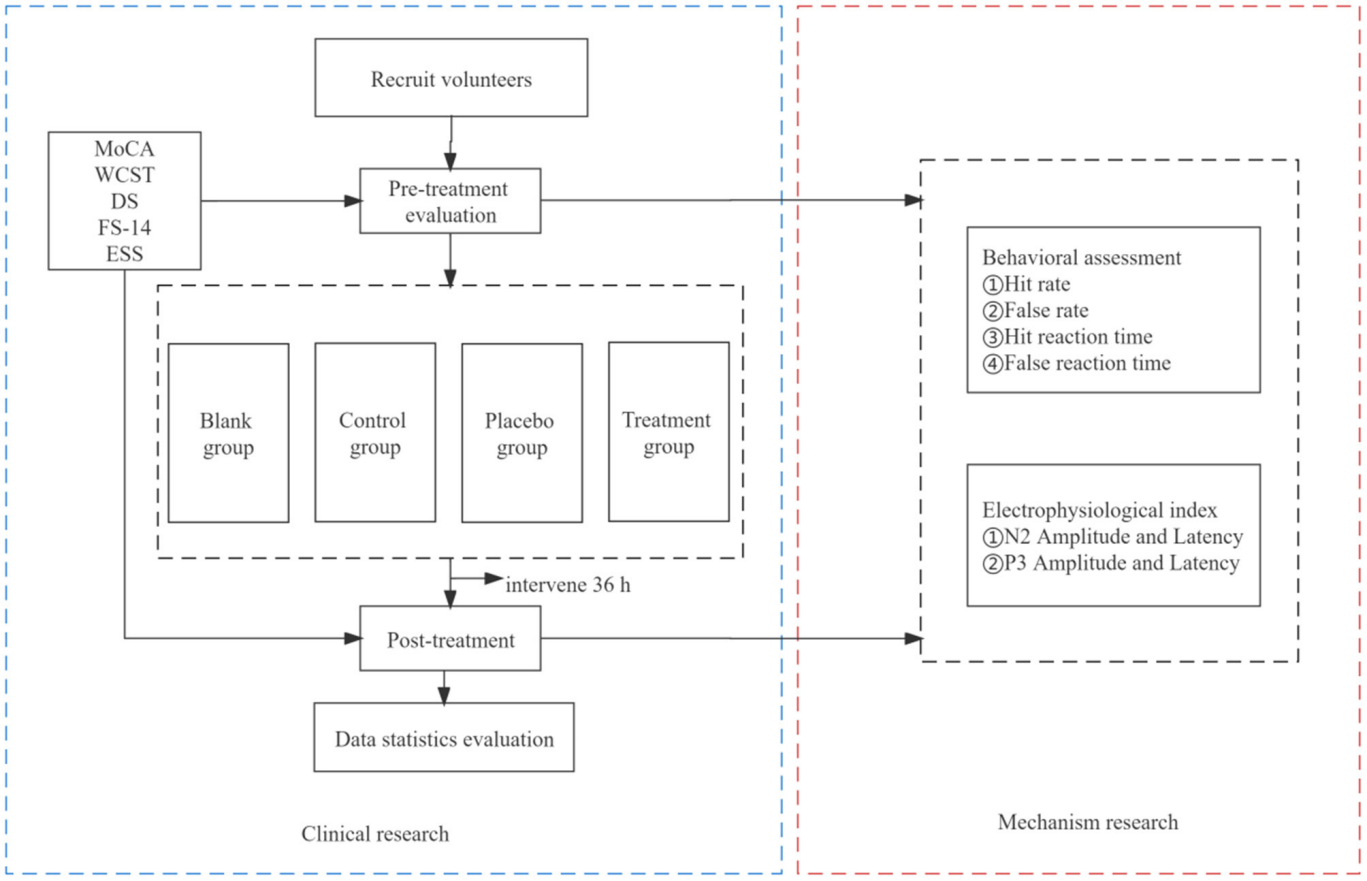
Trial work plan. The primary outcome measure will be MoCA. The remaining outcome measures will be for secondary outcomes.

Twelve hours after the completion of the trial, a follow-up health checkup will be performed, and the participants will be asked if they are experiencing any physical discomfort. The trial work plan is summarized in Figure 2.

### Data Management and Monitoring

Both paper files and electronic documents will be preserved for at least 5 years after publication. If readers have any questions, they can contact the corresponding author for access to the original data. Patient information will remain anonymous, including name, ID number, and telephone number. The protocol will be reviewed and revised by experts in acupuncture, emergency, methodology, and statistics. We will perform a pre-specified standard operating procedure, which includes screening patients, improving relevant inspection, acupuncture, filling out the CRF, assessing outcomes, and data management. Outcome assessments, completion of case report forms and data management will be closely supervised. We will be monitored by the IEC of the Third Affiliated Hospital of Henan University of Chinese Medicine, and it is independent from the investigators and sponsor which will audit trial conduct every 12 months. Any modifications and corrections to operation procedures will be fully documented using a breach report form, monitored, and submitted to the directors of the ethics committee and China Clinical Trial Registration.

### Data Analysis

We will use SPSS software version 21.0 (IBM Corp, Armonk, New York, US) to perform data analysis. Demographic and baseline data will be analyzed with standard descriptive statistics. Data will be presented as the mean ± standard deviation (SD). A repeated measure ANOVA is employed to analyze the electroacupuncture effects and the time effects on the behavioral data. The repeated measure ANOVA is also used for the analysis of ERP indices. ANOVAs are performed on the N2 and P3 components of the scalp electrodes in the Go/No-Go task. The accepted level of significance for all analyses will be *P* < 0.05.

### Ethics and Dissemination

All candidates who agree to participate and who meet all of the inclusion criteria and none of the exclusion criteria will be provided an informed consent form to provide them with full understanding of what the study participation will entail and the potential risks. Participants have the right to discontinue participation at any time. Data will be used in the aggregate only, and no identifying characteristics of individuals will be published or presented. In the consent form, the participants will be asked if they agree to the use of their data, should they choose to withdraw from the trial. The participants will also be asked for permission for the research team to share relevant data with people from the regulatory authorities, where relevant. This trial does not involve collecting biological specimens for storage. The study conforms to the principles of the Declaration of Helsinki. Ethical approval has been obtained from the IEC of the Third Affiliated Hospital of Henan University of Chinese Medicine. The trial protocol has been registered at the Chinese Clinical Trial Registry. The results will be disseminated through journal articles, a master's thesis, or conference presentations.

## Discussion

The purpose of this trial is to assess the impact of acupuncture on the management of brain cognition control. It is obvious that cognitive function is impaired after sleep deprivation. We intend to compare the effects of time (baseline and 36-h ASD) and intervention (electroacupuncture, sham electroacupuncture and non-electroacupuncture treatment) on executive brain function using a visual Go/No-Go task with simultaneous EEG recordings. Acupuncture is a non-toxic, economical intervention with minimal adverse effects (32) that has been shown to be effective after the therapy (11).

There are several methodological limitations to this study: (1) Due to the characteristics of acupuncture is the non-blinding of the acupuncturist. (2) Only young male subjects will be included; so, the findings may not be generalizable to women and older people. (3) The sample size is small. Despite these limitations, we will use rigorous methodology in this study, and we hope that the trial will help provide new insights into the value of acupuncture and evidence of ERP in executive brain function.

## Trial Status

Recruiting will start in February 2022. The current protocol is version 1 of 27-12-2021. Patient recruitment is estimated to be completed around June 2022.

## Ethics Statement

The studies involving human participants were reviewed and approved by The Third Affiliated Hospital of Henan University of Traditional Chinese Medicine. The patients/participants provided their written informed consent to participate in this study.

## Author Contributions

XC is the principal investigator, conceived the study and led the proposal, protocol development, study design, and methodology. HL and MW drafted the manuscript and performed the trial registration. YW will perform electroacupuncture operation and provided critical revision of the manuscript. CX designed the statistical analysis. PL, RZ, and ZL participated in the data collection. All authors have read and approved the final manuscript.

## Funding

This trial was funded by National Natural Science Foundation of China Funded project plan (No. 81704183), the Traditional Chinese Medicine Administration of Henan Province (Nos. 2022ZYZD12 and 2018ZY2009), and the Construction Project of Chinese Medicine in Henan Province (STG-ZYX02-202111). The sponsor and funder have not participated in the study design, data collection and management, analysis, or interpretation of the data, the writing of the report, the decision to publish the report, or the preparation of the manuscript. The funder does not have ultimate authority over any of these activities.

## Conflict of Interest

The authors declare that the research was conducted in the absence of any commercial or financial relationships that could be construed as a potential conflict of interest.

## Publisher's Note

All claims expressed in this article are solely those of the authors and do not necessarily represent those of their affiliated organizations, or those of the publisher, the editors and the reviewers. Any product that may be evaluated in this article, or claim that may be made by its manufacturer, is not guaranteed or endorsed by the publisher.

## Abbreviations

ASD: acute sleep deprivation
ERPs: event-related potentials
EHQ: Edinburgh Handedness Questionnaire
SCL-90: Symptom Checklist-90
PSQI: Pittsburgh Sleep Quality Index
MoCA: Montreal Cognitive Assessment
WCST: Wisconsin Card Sorting Test
DS: Digit Span of the Wechsler Adult Intelligence Scale
WAIS: Wechsler Adult Intelligence Scale
ESS: Epworth Sleepiness Scale
FS-14: Fatigue Scale-14
ANOVA: analysis of variance
IEC: Institutional Ethics Committee
EEG: electroencephalogram.
